# Giant Meckel’s Diverticulum as a Cause of Post-traumatic Hemoperitoneum in a 25-Year-Old Male: An Extremely Rare Phenomenon

**DOI:** 10.7759/cureus.36688

**Published:** 2023-03-26

**Authors:** Muhammad Nasir, Ali Gohar, Haseeb Mehmood Qadri, Fahad Qayyum, Rabia Rehman, Hassan Chaudhry, Kashif Iqbal

**Affiliations:** 1 General Surgery, Lahore General Hospital, Lahore, PAK; 2 General Surgery, Jinnah Hospital, Lahore, PAK; 3 Surgery, Sharif Medical City Hospital, Lahore, PAK; 4 Surgery, Rashid Latif Medical College, Lahore, PAK; 5 Surgery, Azra Naheed Medical College, Lahore, PAK

**Keywords:** chatgpt, hemoperitoneum, diverticulectomy, blunt abdominal injury, explorative laparotomy, giant meckel’s diverticulum

## Abstract

Following the "rule of 2", Meckel's Diverticulum (MD) is 2 inches or 5cm long. However, we report the case of an extremely large MD. To the best of our elucidated literature search, it is the first case of Giant Meckel's Diverticulum (GMD) from Pakistan presenting with post-traumatic hemoperitoneum.

A 25-year-old Pakistani male presented to a surgical emergency with a two-hour history of generalized abdominal pain after blunt abdominal trauma. An exploratory laparotomy was carried out due to the deranged hemodynamic parameters and free fluid in the abdominopelvic cavity, revealing a 35 centimeters long MD with a bleeding vessel on its tip. Diverticulectomy with the repair of a small intestinal defect was performed after the evacuation of 2.5 liters of clotted blood. Histologic evaluation revealed ectopic gastric tissue. He had an uneventful post-operative stay and was discharged home.

The current English scientific literature has adequate case reports documenting the complications of perforation, intestinal obstruction, and diverticulitis of Meckel's Diverticulum (MD) of normal length. However, this case report highlights the significance of an MD with an abnormal length which put the patient's life at risk of death in the setting of normal intra-operative anatomy of all other abdominal organs.

## Introduction

The most common congenital anomaly of the human gastrointestinal tract is the presence of Meckel's diverticulum (MD) [1]. MD is a true intestinal diverticulum created by the incomplete obliteration of the vitellointestinal duct [2]. It is commonly associated with the Fibonacci sequence called the "rule of 2s", occurring in two percent of the population, with a peak at two years of age, located two-feet proximal from the ileocecal valve and two inches in length. It can have two types of heterotopic tissues, mainly gastric and occasionally pancreatic, a twice male preponderance and becoming symptomatic in only 2-4% of individuals [3,4].

Most individuals with MD remain asymptomatic throughout their lifetime. However, the most common symptoms include fever, vomiting, and abdominal pain [2]. The clinical features associated with its symptomatic form can be confused with acute appendicitis, gastroenteritis, and diverticular colon disease [1]. Although computed tomography (CT) is a superior imaging method in adult patients with an emergency presentation, a 99m-technetium-labeled pertechnetate scan is used to localize ectopic gastric mucosa in MD [3].

An MD over 5 cm long is classified as a Giant Meckel's Diverticulum (GMD) [3]. The reported rate of complications is 4-40% [5]. In pediatric patients, gastrointestinal bleeding and obstruction are the commonly encountered complications [2]. While in adults, intestinal obstruction is the most frequently reported clinical presentation, mainly due to intussusception or intestinal volvulus and rarely due to diverticulitis, diverticular torsion, or Littré's hernia (abdominal wall hernia that involves the Meckel's diverticulum) [4]. Diverticulectomy is done to manage symptomatic MD; however, it is controversial for asymptomatic and incidentally found Meckel's diverticulum [3].

After a comprehensive literature search on PubMed, Scopus, and Google Scholar, we deduce that this case is the first to be reported from Pakistan describing Giant Meckel's Diverticulum (GMD). Many cases have been reported of the complications mentioned above of GMD worldwide. However, a case report showing GMD as a cause of major post-traumatic hemorrhage and a size of 35cm has not been documented.

## Case presentation

**Figure 1:**
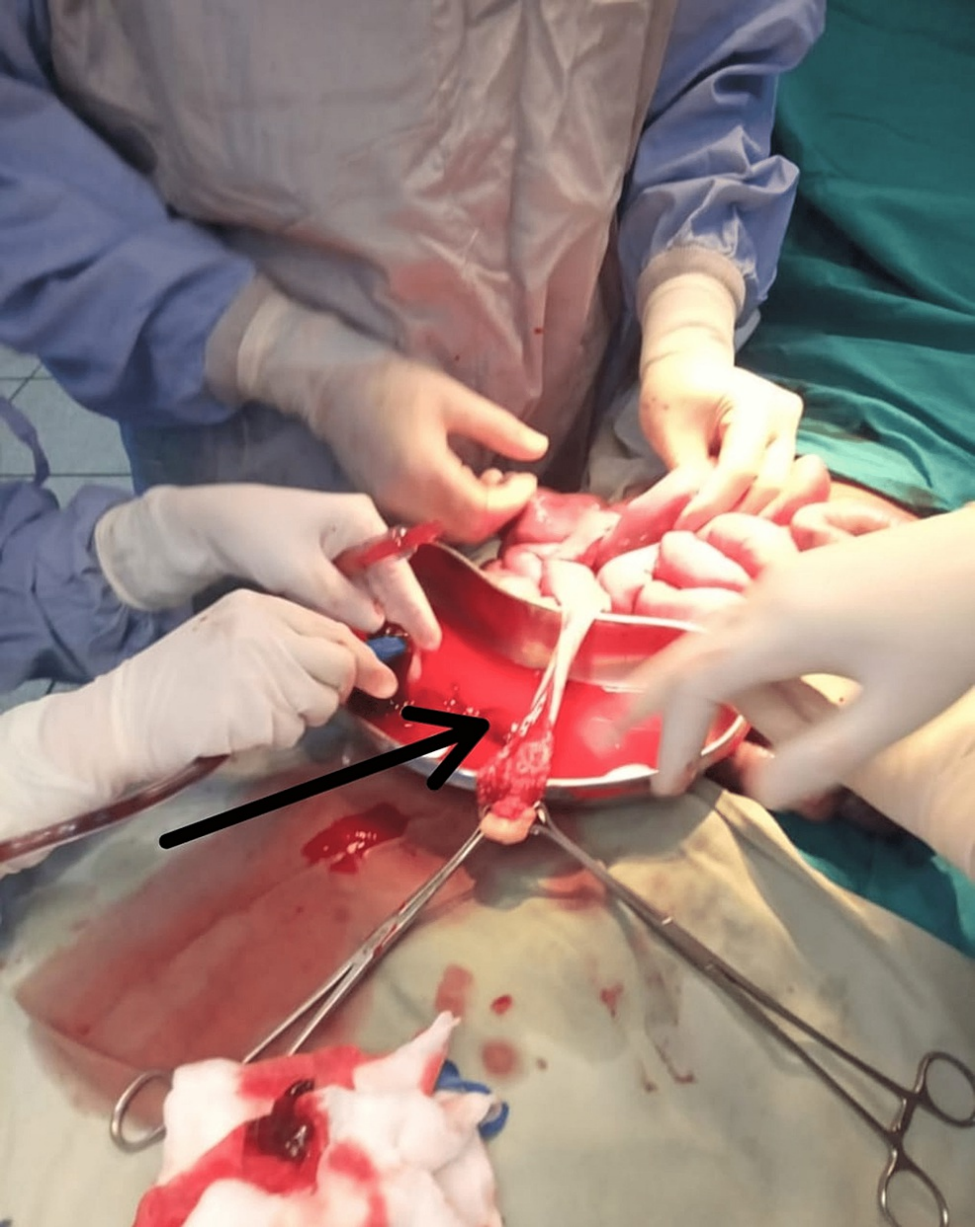
Intra-operative 35cm long Giant Meckel's Diverticulum held by Babcock's forceps.

A 25-year-old male resident of Lahore, a junk-yard driver by profession with having body mass index (BMI) of 22.5 kg/m^2^, presented to the surgical emergency of Lahore General Hospital, Lahore, Pakistan, in January 2021 with a complaint of generalized abdominal pain for the past 2 hours. He underwent blunt abdominal trauma in a road traffic accident. The abdominal pain was sudden in onset without any aggravating or relieving factors. It was associated with nausea and a single episode of food-stained vomitus. There was no significant past medical or surgical history. The presenting vitals were a pulse rate (PR) of 120 beats per minute, blood pressure (BP) of 90/60 mmHg, respiratory rate (RR) of 16 per minute, and oxygen saturation of 96% at room temperature and pressure. Upon examination, the abdomen was distended and tender, with sluggish bowel sounds. Clinical examination for other systems was unremarkable. All baseline investigations were normal, except complete blood count (CBC) showing a neutrophilic leukocytosis of 18.8 ×10^9^/L. Our provisional diagnosis was peritonitis secondary to enteric perforation. Ultrasound abdomen and pelvis showed a "moderate amount of fluid in the abdominopelvic cavity". The consultant on call decided to explore the patient owing to the deranging hemodynamic parameters of the patient and ultrasound findings. A midline exploratory laparotomy was performed. Per-operatively, 2.5 liters of clotted blood in the abdominopelvic cavity was evacuated. Bowel contents were explored for the source of bleeding. A large Meckel's diverticulum was located approximately 76cm proximal to the ileocecal junction, and a bleeding vessel was present at the tip of Meckel's diverticulum. The length of MD was 35cm (Figure 1).

**Figure 2:**
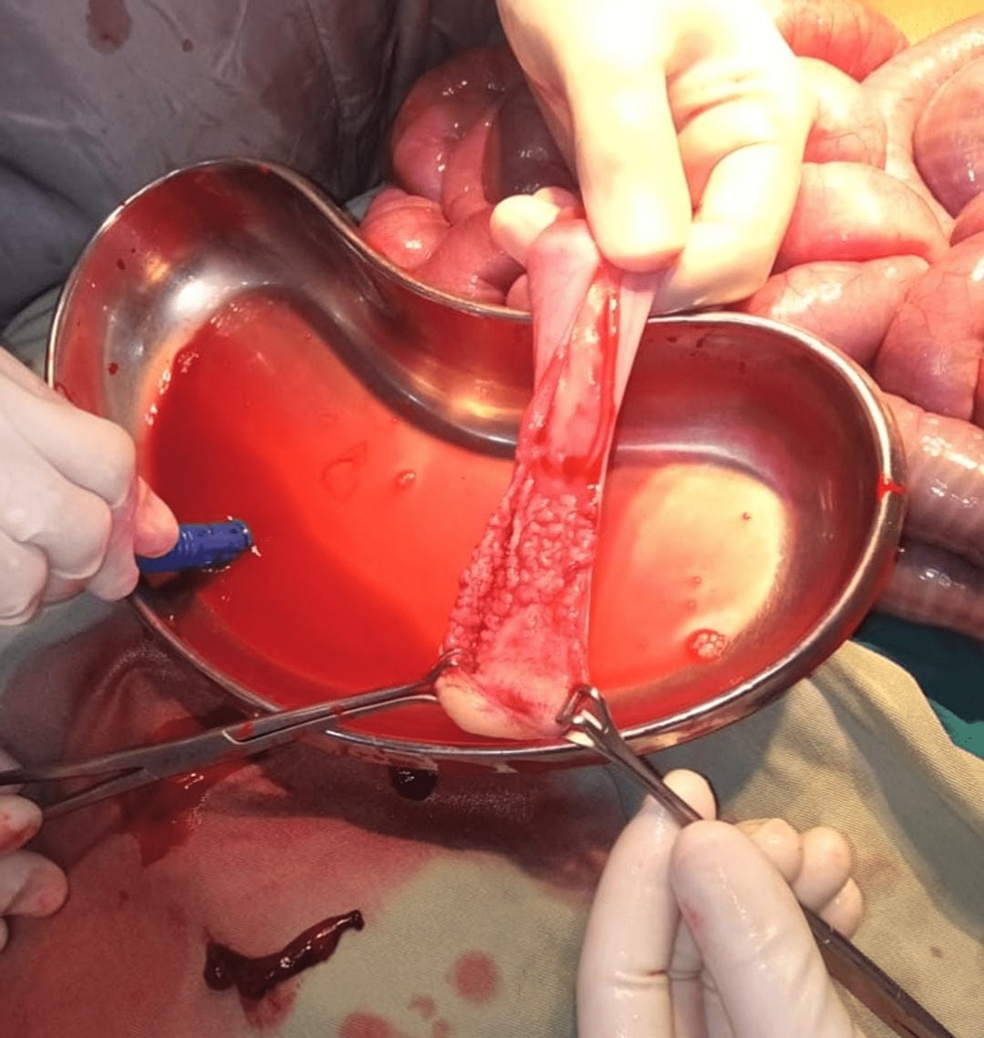
Before diverticulectomy: The intra-luminal appearance of Giant Meckel's Diverticulum resembling gastric mucosa can be appreciated.

All abdominal and pelvic organs were normal. The bleeding vessel present on the apex of MD was ligated, and diverticulectomy with the repair of the defect in the small bowel was done with absorbable sutures. The Giant Meckel's Diverticulum (GMD) was incised to view its gross intra-luminal appearance, which resembled gastric rugae (Figure 2).

A closed-drain system was placed in the pelvic cavity. There were no intra-operative complications, recovery from general anesthesia was smooth, and the patient was shifted to a stable male bay. The patient had an in-hospital stay of eight days due to post-operative fever complaints. We found basal atelectasis as the cause of fever after clinical examination and chest X-ray (CXR), which was managed via prophylactic antibiotic cover, incentive spirometry, and chest physiotherapy. Meanwhile, his drain output decreased gradually and was removed after 48 hours. His oral feed was started with liquids on the sixth post-operative day, followed by semi-solids on the day of discharge. He was discharged home smoothly. The histopathology report confirmed Giant Meckel's Diverticulum (GMD) with the gastric mucosa. We followed the patient on two different visits. There were two visits in the first month, followed by one per month for the next five months. Each follow-up visit included the patient's clinical examination and wound healing and care instructions. The patient is living a healthy life these days.

## Discussion

Less than 0.5% of vitelline anomalies can be classified formally as Giant Meckel's Diverticula (GMD). Of the cases, 80% are asymptomatic, and the lifetime risk of developing a complication is 6.4% [6]. GMD has further classified into type 1 elongated and type 2 ovoid forms, depending on their gross morphology. Some authors reserve the term 'giant' for type 2 diverticula [6].

We document a unique case of hemorrhagic GMD. MD is preponderant among males; our patient was also a young male in his third decade. A case report presented by Srilankan authors reported a 20-year-old male laborer with spontaneous axial torsion of GMD [1]. On the contrary, our patient had a history of blunt trauma directed toward the vasculature of GMD. The clinical findings are usually dominated by lower abdominal pain, fever, and vomiting with right iliac fossa tenderness on abdominal examination [7]. The systemic examination in our patient also revealed a distended, tender abdomen with sluggish bowel sounds on auscultation.

Ultrasound abdomen is usually the first diagnostic tool showing small bowel obstruction with air-fluid levels [1]. Our patient also underwent an ultrasound abdomen revealing a moderate amount of abdominopelvic fluid. A similar study seeking the cause of anemia in a young male published in England highlights that 99m technetium-pertechnetate is highly reserved for the affected pediatric population. At the same time, computerized tomography (CT) scan is advocated for young adults [6]. The former has a higher sensitivity (85-90%) in the pediatric population than in adult (60%) patients [5]. We refrained from performing CT Abdomen due to the clear free fluid in the abdominopelvic cavity and the deteriorating hemodynamic parameters of our patient. Existing English scientific literature shows various radiographic signs in patients with GMD, like the triradiate fold pattern, whirlpool sign, mucosal triangular plateau, opacification, and gastric rugal pattern [3,6-8].

Khan et al. propose that diverticulectomy, wedge resection, or surgical resection, whether via laparoscopy or laparotomy, are the currently available management options. Various factors, i.e., the presence or absence of ectopic tissue, its location, and the integrity of the diverticular base, govern the optimum approach used [9]. Diverticulectomy, with or without resectioning a portion of the small intestine, is the mainstay treatment in symptomatic GMD, and associated complications are addressed depending on their nature [1,3,7]. Our approach to managing the patient was analogous in this regard. We excised the 35cm long GMD and sutured the defect in the small bowel with Vicryl 3/0. Such lengthy MD has yet to be reported in English scientific literature [1,3,4,6-8].

MDs are lined by heterotopic mucosa in 60% of cases, with the gastric type being the most common (62%), followed by pancreatic type among 6% of affected individuals. The remaining cases combine the above two and intestinal-type mucosa 5% [5]. Likewise, the histo-pathology of resected GMD was concordant with gastric-type mucosa in our patient.

**Figure 3:**
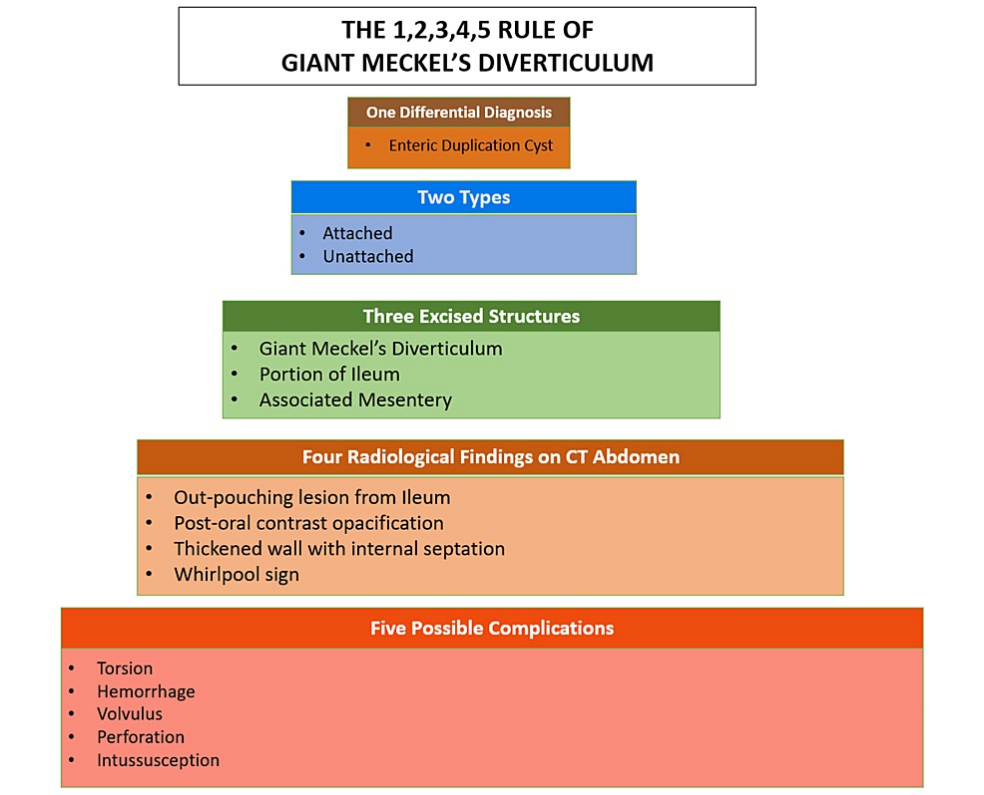
The 1, 2, 3, 4, 5 Rule of a Giant Meckel's Diverticulum (This figure is developed by the article's authors purely and wholly after a detailed literature study of the cited papers).

Much is known about Meckel's Diverticulum, but we have attempted to ascertain the most common differential diagnosis, types, resected structures, radiographic findings, and possible complications of a Giant Meckel's Diverticulum (Figure 3).

## Conclusions

Post-traumatic hemoperitoneum secondary to Giant Meckel's Diverticulum (GMD) is a rare clinical phenomenon. Operative intervention remains the mainstay of management in symptomatic patients. Existing literature highlights the diagnostic limitation of ultrasound and computerized tomography scans in diagnosing this clinical entity. These investigatory modalities are insignificant until there are signs of associated complications, and a 99m-labeled-technetium pertechnetate scan does not help diagnose if MD is without ectopic mucosa.

We emphasize that though the occurrence of Meckel's Diverticulum (MD) is rare and Giant Meckel's Diverticulum (GMD) is even rarer, it must be considered as an important differential diagnosis in patients who do not follow the "rule of 2's" for their age but present with abdominal pain. Despite lacking diagnostic modalities and rare findings, the spotlight must be put on Meckel's Diverticulum to reduce the morbidity and mortality associated with it or its complications.

## Figures and Tables

**Figure 4:**
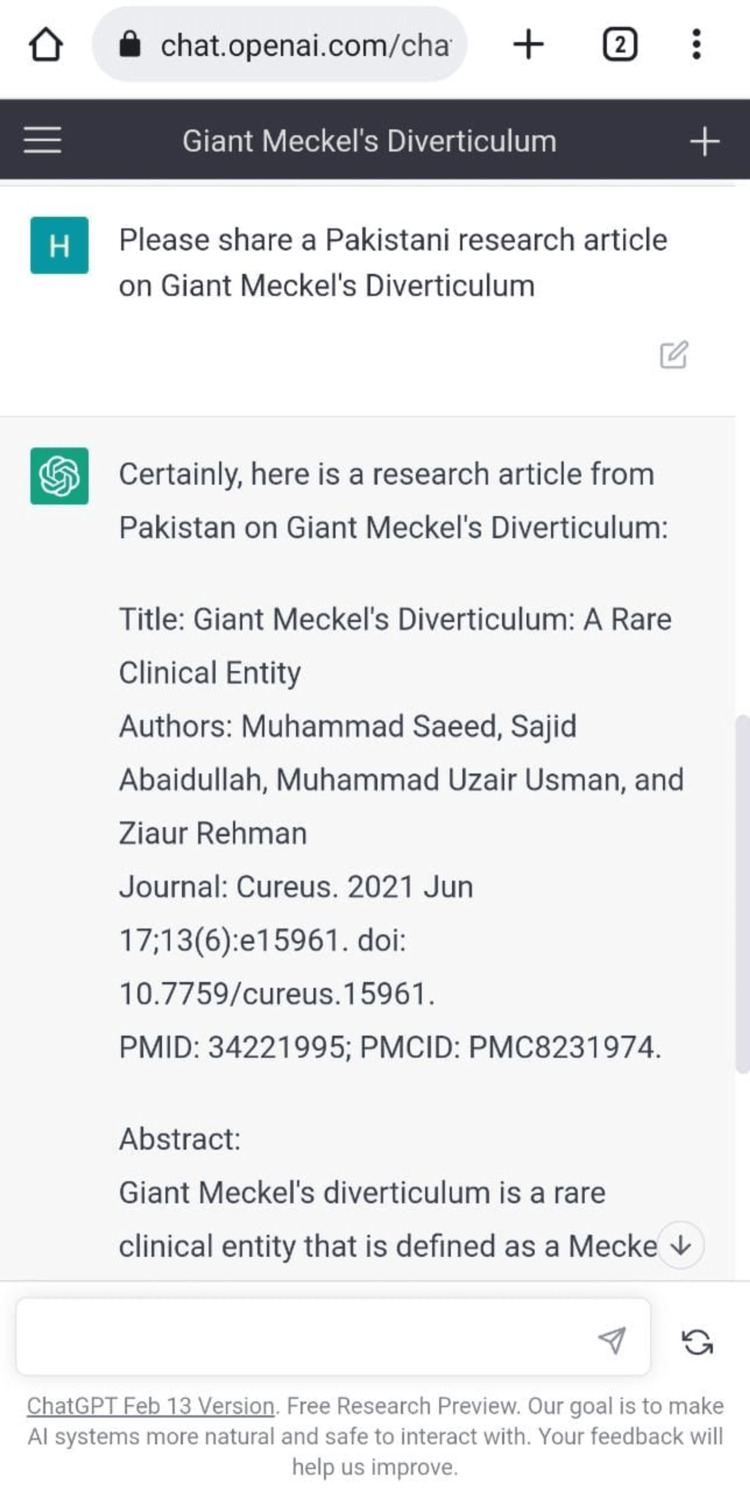
Literature Search of a Pakistani Case Report on Meckel's Diverticulum using ChatGPT.

**Figure 5:**
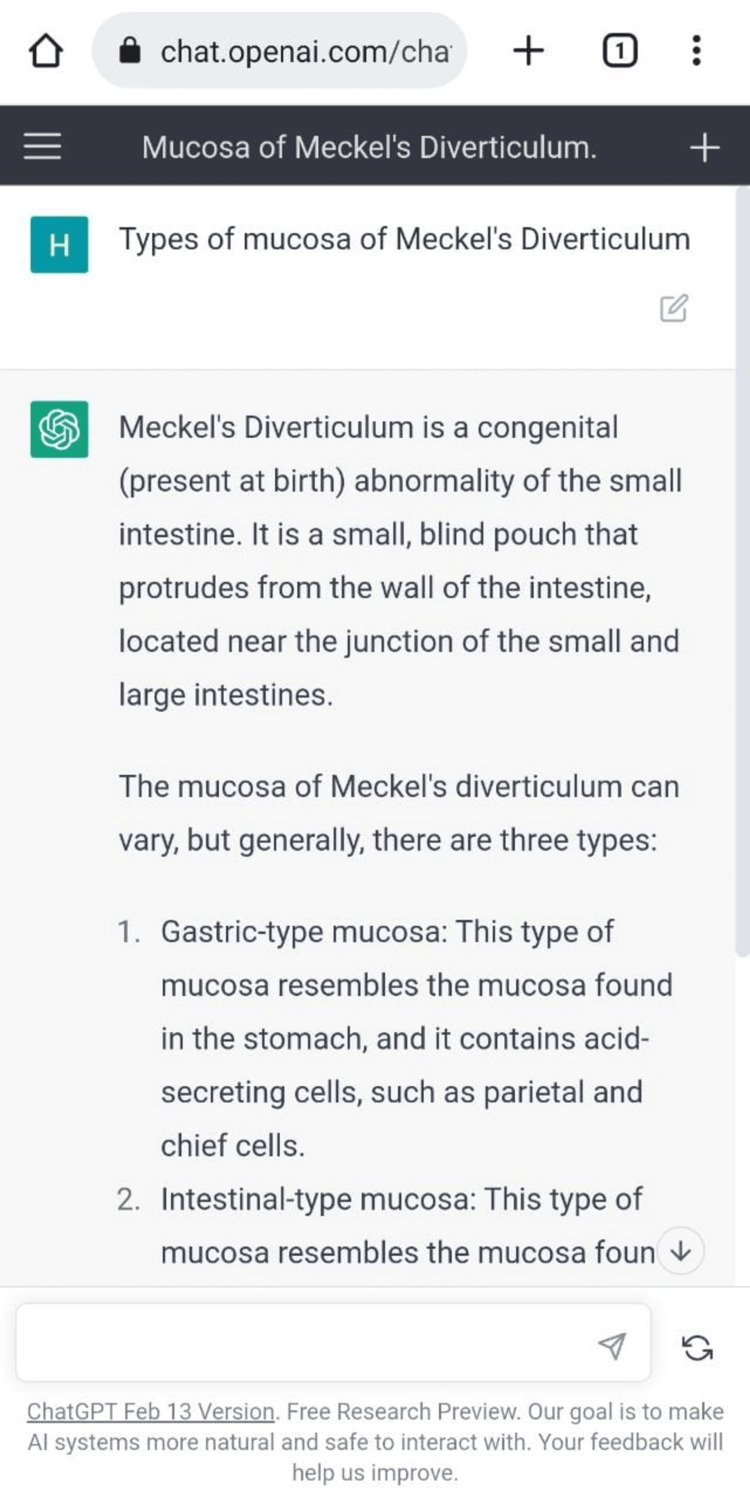
Literature Search on Types of Ectopic Tissue Found in Meckel's Diverticulum using ChatGPT.

**Figure 6:**
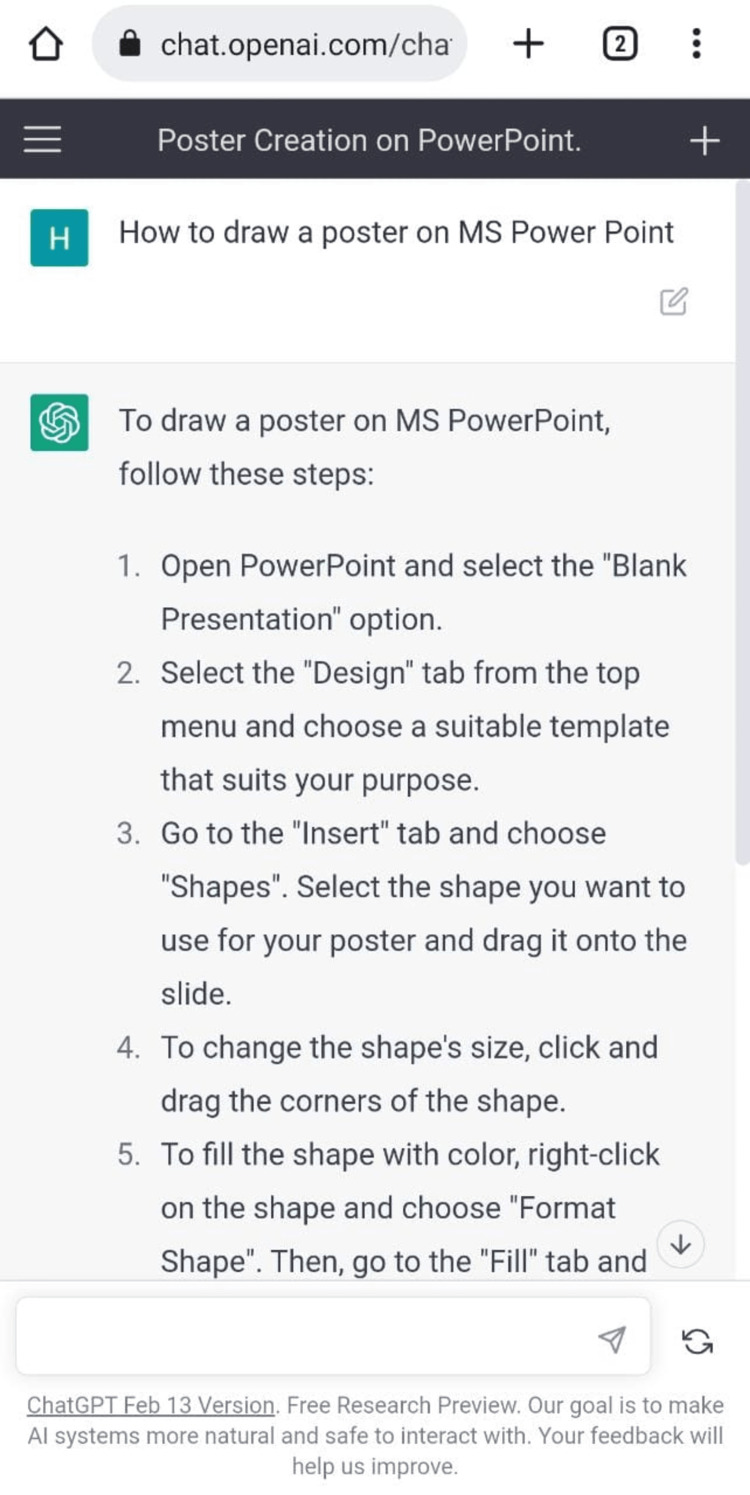
Elaborated Instructions on How to Create a Poster by ChatGPT.

## Appendices

I am thankful to the developers of ChatGPT AI Open Systems. ChatGPT is mind-blowing!

I extended my literature search using ChatGPT and found a Pakistani study on the same topic (Figure 4).

I was confused about the types of ectopic tissue found in Meckel's Diverticulum. My ChatGPT application helped me clear the relevant concepts, and I searched for more articles on the same topic using ChatGPT (Figure 5).

I was not an expert at creating posters. I created Figure 3 of our case report following the instructions of ChatGPT. ChatGPT is my mentor now! (Figure 6)

MN and HMQ planned, designed, and supervised the study; MN, HMQ, and AG did a literature search; AG, HMQ, FQ, HFC, RR, and KI studied the literature comprehensively; AG, HMQ, FQ, HFC, RR, and KI did manuscript drafting; MN, HMQ, and FQ edited and completed the final manuscript; HMQ and HFC provided the image support, HMQ, AG, RR, and KI formatted the article, MN and HMQ proof-read the article. All authors agree to the final version of the submitted article and have a consensus on the authors' positions within the article.
